# Prediction of Next Glucose Measurement in Hospitalized Patients by Comparing Various Regression Methods: Retrospective Cohort Study

**DOI:** 10.2196/41577

**Published:** 2023-01-31

**Authors:** Andrew D Zale, Mohammed S Abusamaan, John McGready, Nestoras Mathioudakis

**Affiliations:** 1 Division of Endocrinology, Diabetes & Metabolism Department of Medicine Johns Hopkins University School of Medicine Baltimore, MD United States; 2 Department of Biostatistics Johns Hopkins Bloomberg School of Public Health Baltimore, MD United States

**Keywords:** hospital, glucose, inpatient, prediction, regression, machine learning

## Abstract

**Background:**

Continuous glucose monitors have shown great promise in improving outpatient blood glucose (BG) control; however, continuous glucose monitors are not routinely used in hospitals, and glucose management is driven by point-of-care (finger stick) and serum glucose measurements in most patients.

**Objective:**

This study aimed to evaluate times series approaches for prediction of inpatient BG using only point-of-care and serum glucose observations.

**Methods:**

Our data set included electronic health record data from 184,320 admissions, from patients who received at least one unit of subcutaneous insulin, had at least 4 BG measurements, and were discharged between January 1, 2015, and May 31, 2019, from 5 Johns Hopkins Health System hospitals. A total of 2,436,228 BG observations were included after excluding measurements obtained in quick succession, from patients who received intravenous insulin, or from critically ill patients. After exclusion criteria, 2.85% (3253/113,976), 32.5% (37,045/113,976), and 1.06% (1207/113,976) of admissions had a coded diagnosis of type 1, type 2, and other diabetes, respectively. The outcome of interest was the predicted value of the next BG measurement (mg/dL). Multiple time series predictors were created and analyzed by comparing those predictors and the index BG measurement (sample-and-hold technique) with next BG measurement. The population was classified by glycemic variability based on the coefficient of variation. To compare the performance of different time series predictors among one another, *R*^2^, root mean squared error, and Clarke Error Grid were calculated and compared with the next BG measurement. All these time series predictors were then used together in Cubist, linear, random forest, partial least squares, and k*-*nearest neighbor methods.

**Results:**

The median number of BG measurements from 113,976 admissions was 12 (IQR 5-24). The *R^2^* values for the sample-and-hold, 2-hour, 4-hour, 16-hour, and 24-hour moving average were 0.529, 0.504, 0.481, 0.467, and 0.459, respectively. The *R^2^* values for 4-hour moving average based on glycemic variability were 0.680, 0.480, 0.290, and 0.205 for low, medium, high, and very high glucose variability, respectively. The proportion of BG predictions in zone A of the Clarke Error Grid analysis was 61%, 59%, 27%, and 53% for 4-hour moving average, 24-hour moving average, 3 observation rolling regression, and recursive regression predictors, respectively. In a fully adjusted Cubist, linear, random forest, partial least squares, and k*-*nearest neighbor model, the *R^2^* values were 0.563, 0.526, 0.538, and 0.472, respectively.

**Conclusions:**

When analyzing time series predictors independently, increasing variability in a patient’s BG decreased predictive accuracy. Similarly, inclusion of older BG measurements decreased predictive accuracy. These relationships become weaker as glucose variability increases. Machine learning techniques marginally augmented the performance of time series predictors for predicting a patient’s next BG measurement. Further studies should determine the potential of using time series analyses for prediction of inpatient dysglycemia.

## Introduction

### Background

Current practice guidelines recommend scheduled insulin therapy for most hospitalized patients with diabetes or hyperglycemia [1]. Insulin is a narrow therapeutic index medication and has been linked to hypoglycemia in up to 28% of patients [2]. As inhospital hypoglycemia has been associated with increased patient mortality and poor admission outcomes, improving glycemic control remains of utmost importance to minimize the burden of hypoglycemia [3-6]. Safely and effectively prescribing insulin in the hospital can be challenging owing to the presence of dynamic factors, such as steroid doses, infection, renal status, and diet, that can influence glucose homeostasis in ways that may be difficult to predict [7-10].

In an attempt to combat hypoglycemia, continuous glucose monitors (CGMs) were developed to measure the glucose concentration on the time scale of minutes [11-13]. Sparacino et al [14] determined that a hypoglycemic event could be predicted with a prediction horizon of 30 minutes using a first-order autoregressive model with CGM data. Using more advanced machine learning methods in addition to the time series data provided by CGMs led to improved 5-minute and 30-minute blood glucose (BG) predictions [15,16]. A recent review suggests that deep learning and artificial neural network models perform better for glucose prediction than probabilistic and static models [17]. Notably, though, the addition of physiological parameters, such as insulin dosing, only marginally improved the prediction of hypoglycemia in certain predictions [18]. Despite the promise that CGMs have offered to improve inhospital glucose control [1], CGMs are not widely used in the hospital setting and are not currently the standard of care for inpatient glucose management [19-21]. As point-of-care (POC) finger-stick BG testing (typically 4-6 times daily) is the standard in hospitalized patients, we aimed to understand whether the tools used for CGM prediction could be applied for prediction using less frequent glucose data points [22].

### Objectives

The objective of this study was to compare different regression windows and machine learning approaches for prediction of the next BG measurement in hospitalized patients using only POC and serum glucose data. As previous research has suggested that self-monitoring BG underpredicts low and high BG indices owing to the sparser data points compared with CGM, there is a compelling need to improve on predictive accuracy using POC BG measurements [23]. Most published machine learning prediction models in the inpatient setting have been developed for binary [24-34] (ie, hypoglycemia vs not) or categorical [35,36] glucose outcomes (ie, controlled, hyperglycemic, and hypoglycemia) rather than a continuous glucose outcome [37] (ie, glucose value) [38]. We have previously published models that seek to predict hypoglycemia by considering BG as a categorical variable [24,35]. Although those models showed promising early results, we are seeking to develop an algorithm that quantitatively predicts a patient’s next BG reading by beginning to consider BG as a quantitative variable, similar to the methodology used in CGMs, with the caveat that inpatient BG measurements occur less frequently and with more variability than those of CGMs. For prediction of glucose as a continuous outcome, previous studies have highlighted that moving average (MA) and other time series models are effective when using CGM data [39-41]. Thus, we sought to use time series analytic tools to quantitatively predict a patient’s next BG measurement in the hospital.

## Methods

### Data Set

This was a retrospective cohort study derived from electronic health record data obtained from 5 hospitals within the Johns Hopkins Health System. Admissions were included if patients received at least one unit of either subcutaneous or intravenous insulin and had at least 4 BG measurements during the admission. Among 118,734 hospitalized patients discharged between January 1, 2015, and May 31, 2019, there were a total of 4,538,510 serum or POC BG measurements. Data extraction and processing methodology for this data set has been previously described [28]. As previously reported, the characteristics of patients included from the 5 hospitals differed with respect to age (median 59-74 years), sex (45.7%-51.2% male), and race (53.3%-64.5% White). The percentage of patients who were prescribed insulin at home in each of the 5 hospitals ranged from 7.6% to 14.5% [35].

As our interest was to learn about prediction in noncritical care settings where glucose measurements are typically obtained 4-6 times daily, we excluded admissions in which the patient received intravenous insulin or was admitted to the intensive care unit (typically hourly glucose checks), or had a BG measurement in which both the preceding and succeeding BG measurements were within ≤90 minutes. The rationale for this last exclusion criterion was 2-fold: first, the typical shortest interval between BG checks for most patients in a nonintensive care unit setting is 3-4 hours (between meals and at bedtime, or every 4-6 hours for select patients), and second, successive BG measurements correlate with one another, which would overestimate model performance. Furthermore, because the longest interval between 2 finger-stick BG measurements in hospitalized patients is typically 10 hours (window between 10 PM bedtime and 8 AM finger stick), we excluded any BG reading in which the succeeding BG reading occurred >10 hours after that BG reading. These exclusions decreased our data set from 184,320 admissions to 113,976 admissions.

**Figure 1:**
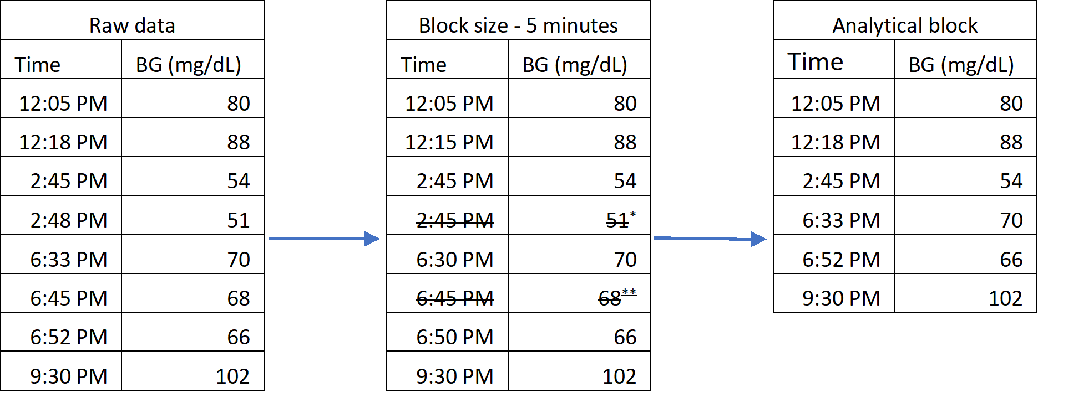
Example of data preprocessing to develop a data set suitable for time series analysis. Preprocessing of the time data required going from standard time (left) to blocks of 5 minutes. Only the first blood glucose (BG) measurement in each block of 5 minutes was included, causing the 51 mg/dL reading at 2:48 to be excluded as a repeat measure of the same hypoglycemic event. In addition, the BG measurement of 68 at 6:45 PM was excluded since the preceding and succeeding BG measurements (at 6:33 PM and 6:52 PM) were both within 90 minutes. In the final data set (right), neither the 6:33 PM nor the 6:45 PM BG measurement were excluded as each of them had at least one adjacent BG measurement separated in time by at least 90 minutes. *Repeat measurement in the same 5-minute block is dropped. **BG measurements in which previous and next reading are both within 90 minutes of index observation is dropped.

For this time series analysis, each BG value was ordered sequentially in 5-minute intervals (Figure 1). For example, a BG measured at 12:16 PM and 12:18 PM would both be grouped in the 12:15 PM 5-minute window. In this case, the second BG value was excluded. We chose the 5-minute window size for 2 reasons. First, a previous study suggested that excluding repeated glucose measurements within a 5-minute interval reduces the chances of overestimation of hypoglycemia owing to repeated measurement of the same hypoglycemic episode [42]. Second, as the statistical software used (Stata; StataCorp LLC) will analyze up to 1000 windows in a time series analysis, the 5-minute interval allows for MA analysis for over 3 days of BG measurements. For the moving regression analyses, we explored various lookback windows by combining 5-minute windows; for example, a 30-minute MA was composed of six 5-minute intervals. In addition, to account for repetitive measurements of the same BG episode, BG measurements that were preceded and succeeded by BG measurements that occurred within 90 minutes each were excluded. In Figure 1, the BG measurement at 6:45 PM was excluded as it was preceded (at 6:33 PM) and succeeded (at 6:52 PM) by BG measurements that occurred within 90 minutes each. After the described exclusions, we retained 2,436,228 BG measurements for correlation analysis and testing with different machine learning algorithms.

### Outcome

The outcome of interest was the predicted value of a patient’s next BG measurement in mg/dL.

### Predictors

The primary predictor of interest was previous BG measurements. All approaches to predicting next BG used some subset of prior BG measurements based either on a prespecified window of time or a prespecified number of such measurements. Secondary predictors considered included sex; age; race; diabetes diagnosis; *nil per os* status; home insulin; home antihyperglycemic medication; glomerular filtration rate; hydrocortisone equivalents on board; and units of insulin on board for basal insulin, combination insulin, concentrated insulin, intermediate acting insulin, rapid-acting insulin, regular insulin, and ultra–long-acting insulin (model B). The data sources and definitions of these variables have been previously described [28].

### Statistical Analysis

All statistical analyses were performed using R statistical software (version 3.6.2; R Foundation for Statistical Computing) and Stata software (version 15.1; StataCorp LLC). Stata software was used for its preloaded functions in time series analysis that allowed for the generation of different time series variables. R statistical software was used to analyze these time series variables via machine learning algorithms. The *Caret* package in R includes functions for data splitting, model tuning via resampling, and summarizing model performance measures. Descriptive statistics were used to summarize the patient population at the index BG observation. Continuous variables were all nonnormally distributed and summarized with medians and IQRs. Categorical variables were summarized with counts and frequencies.

The first predictive approach was based on simple MA models. MA models predict the next glucose value as a simple average of all readings within a specified time window, including the index reading. These analyses were performed with windows of 30 minutes, 1 hour, 1.5 hours, 2 hours, 4 hours, 8 hours, 12 hours, 16 hours, 20 hours, 24 hours, 36 hours, 48 hours, 60 hours, and 72 hours. If there was no additional reading in the MA lookback window, the predicted next BG measurement is the index BG. We created MA values with windows as short as 30 minutes because despite excluding BG readings that had a preceding and succeeding reading within 90 minutes, it was still feasible to have 2 readings within 30 minutes of one another (Figure 1). As the time window used to compute MA increases, the likelihood that any patient has at least one additional BG reading in that window increases, which makes the resulting prediction less correlated to the current BG measurement. MA values were generated in Stata using the base time series analysis functions. These values were stored and used as predictors in machine learning models detailed later in this section.

**Figure 2:**
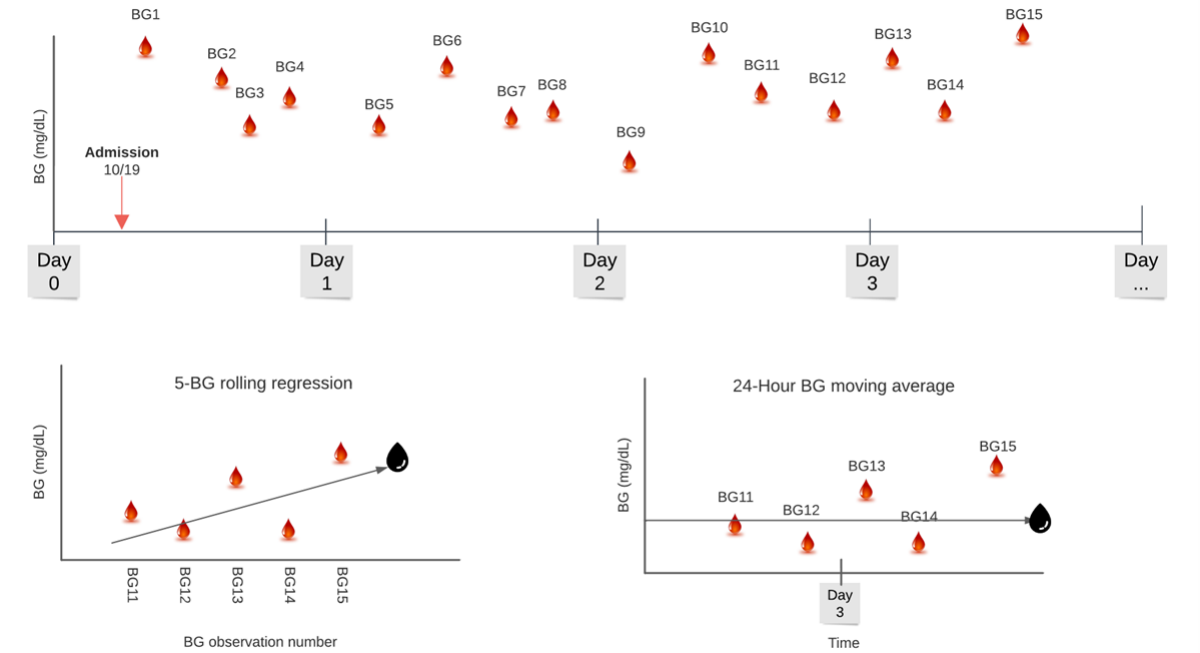
Example calculation of rolling regression and moving average calculation. Top: A patient’s blood glucose (BG) reading is graphically presented with BG value on the y-axis and time of BG reading on the x-axis. Bottom left: Rolling regression removes the temporal component of BG reading. The BG reading number is plotted on the x-axis as a discrete variable, and the BG observation number is plotted on the y-axis. A best-fit line is plotted based on the n BG readings included in the rolling regression, and a prediction (black drop) is made based on where the best-fit line intersects the next discrete BG reading. Bottom right: Moving averages allow for as many BG readings to be included in a given period. All BG readings are equally weighted, and the prediction (black drop) is made based on an unweighted average of all the BG readings in that period.

Rolling regression (RR) was used to predict the next BG measurement from a simple linear regression model estimated from the previous *n* BG measurements. The outcome (y) for these regressions is BG measurement, and the predictor (x) is observation number (1,...,n). Recursive regression, an RR approach where *n* is set to equal the maximum observations (ie, all available BG measurements from admission to index BG value), was also used for next BG prediction. Figure 2 depicts how a patient’s BG measurements are used to calculate a prediction from MA or RR.

*R^2^* estimates were used to quantify the degree of association between the predicted next BG values from the MA and RR analyses and the actual next BG measurement. On the basis of the previous research and descriptions of *R^2^*, we classified *R^2^* values as good if >0.75, acceptable if between 0.50 and 0.75, and inadequate if <0.50 [43]. For this analysis, the data set was further divided into categories of coefficient of variation (CV) to determine if BG measurement lability affected predictive accuracy of different time series predictors: *low glycemic variability* if over the past 24 hours the CV was ≤0.15, *medium glycemic variability* if over the past 24 hours the CV was >0.15 and ≤0.30, *high glycemic variability* if over the past 24 hours the CV was >0.30 and ≤0.45, and *very high glycemic variability* if over the past 24 hours the CV was >0.45. We used this classification system based on previous research, which demonstrated that threshold for high glycemic variability in patients with diabetes is >30%. We chose to further divide the sample to analyze for a dose-response relationship [44-46]. To compare the treatment recommendations in a general hospital population and populations at higher risk of dysglycemia (patients with known diagnosis of type 1 diabetes mellitus [T1DM] or known diagnosis of type 2 diabetes mellitus with basal insulin on board), predictions from the MA and RR analyses were analyzed using Clarke Error Grid analysis. In a Clarke Error Grid, the prediction is plotted on the y-axis and the true measure of the next BG measurement is plotted on the x-axis [47]. Region A represents predictions within 20% of the true values or in the hypoglycemic range when the reference is also <70 mg/dL. Region B contains predictions outside of 20% of the true value but would not lead to inappropriate treatment. Region C contains predictions that lead to unnecessary treatment (predicting hypoglycemia or hyperglycemia when a patient’s BG is controlled). Region D contains predictions that lead to a dangerous failure to detect hypoglycemia or hyperglycemia. Region E contains predictions that misclassify hypoglycemia as hyperglycemia and vice versa.

RR and recursive regression values were generated in Stata using the *asreg* package [48]. The *ega* package in R was used to complete the Clarke Error Grid analysis. The following machine learning methods were also used to estimate the next BG reading: linear regression, partial least squares, Cubist, k-nearest neighbors, and random forest algorithms. The predictors used in each of these models were 30-minute MA, 1-hour MA, 1.5-hour MA, 2-hour MA, 4-hour MA, 8-hour MA, 12-hour MA, 16-hour MA, 20-hour MA, 24-hour MA, 36-hour MA, 48-hour MA, 60-hour MA, 72-hour MA, 3-observation RR, 4-observation RR, 5-observation RR, 10-observation RR, 25-observation RR, 100-observation RR, 500-observation RR, recursive regression, index BG measurement, and previous BG measurements.

To understand the predictive benefit of having non-BG predictors in the machine learning models, we reran the same models listed above including all other patient-level and time-specific predictors in addition to the BG time series measures.

To compare the performance of different machine learning algorithms, a random sample of 10,000 observations was selected for 5-fold cross-validation for each of the 5 methods. Owing to concerns regarding reporting model predictive accuracy with *R*^2^ alone, we also report the median average error and root mean squared error (RMSE) of 5 machine learning models [49]. Machine learning algorithms were developed using the *Caret* R package [50].

### Ethics Approval

The study protocol was approved by the institutional review board of the Johns Hopkins School of Medicine with a waiver of informed consent (IRB00117098).

## Results

### Cohort Characteristics

**Figure 3:**
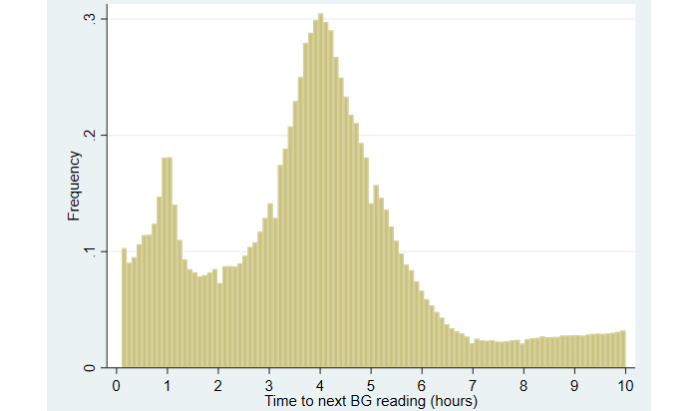
Distribution of time to next blood glucose (BG) reading in hours.

Our cohort includes 2,436,228 BG measurements from 113,976 admissions. Table 1 shows the baseline characteristics of the study population by admission. The population had a median age of 65 (IQR 54-75) years and BMI of 27.8 (IQR 23.6-33.2) kg/m^2^. There was an even sex distribution (57,720/113,976, 50.64% male), and a majority of patients were White (64,517/113,976, 56.61%). The median length of stay for an admission was 5.0 (IQR 3.0-8.9) days. The median average BG admission across an admission was 141 (IQR 117-179) mg/dL; 2.85% (3253/113,976) of the patients had a diagnosis of type 1 diabetes, and 32.5% (37,045/113,976) of the patients had a diagnosis of type 2 diabetes. Figure 3 depicts the distribution of time to next BG reading in hours. The 5th, 25th, 50th, 75th, and 95th percentiles for time to next BG reading were 0.58, 2.48, 3.88, 4.88, and 8.23 hours, respectively. The median number of BG measurements per admission and per hospital day were 12 (IQR 5-24) and 4 (IQR 2-5), respectively.

**Table 1:** Cohort characteristics by admission (N=113,976).

Factor		Value
Age (years), median (IQR)		65 (54-75)
Weight (lbs), median (IQR)		176 (145-213)
BMI (kg/m^2^), median (IQR)		27.8 (23.6-33.2)
**Sex, n (%)**		
	Female	56,256 (49.36)
	Male	57,720 (50.64)
**Race, n (%)**		
	Black	36,371 (31.91)
	Other	13,088 (11.48)
	White	64,517 (56.61)
Length of stay (days), median (IQR)		5.03 (3.01-8.86)
Average admission BG^a^, median (IQR)		141 (117-179)
**Diabetes diagnosis, n (%)**		
	None	72,471 (63.58)
	T1D^b^	3253 (2.85)
	T2D^c^	37,045 (32.5)
	Other	1207 (1.06)

^a^BG: blood glucose.

^b^T1D: type 1 diabetes.

^c^T2D: type 2 diabetes.

### Correlation of Next BG Measurement Predictions

**Figure 4:**
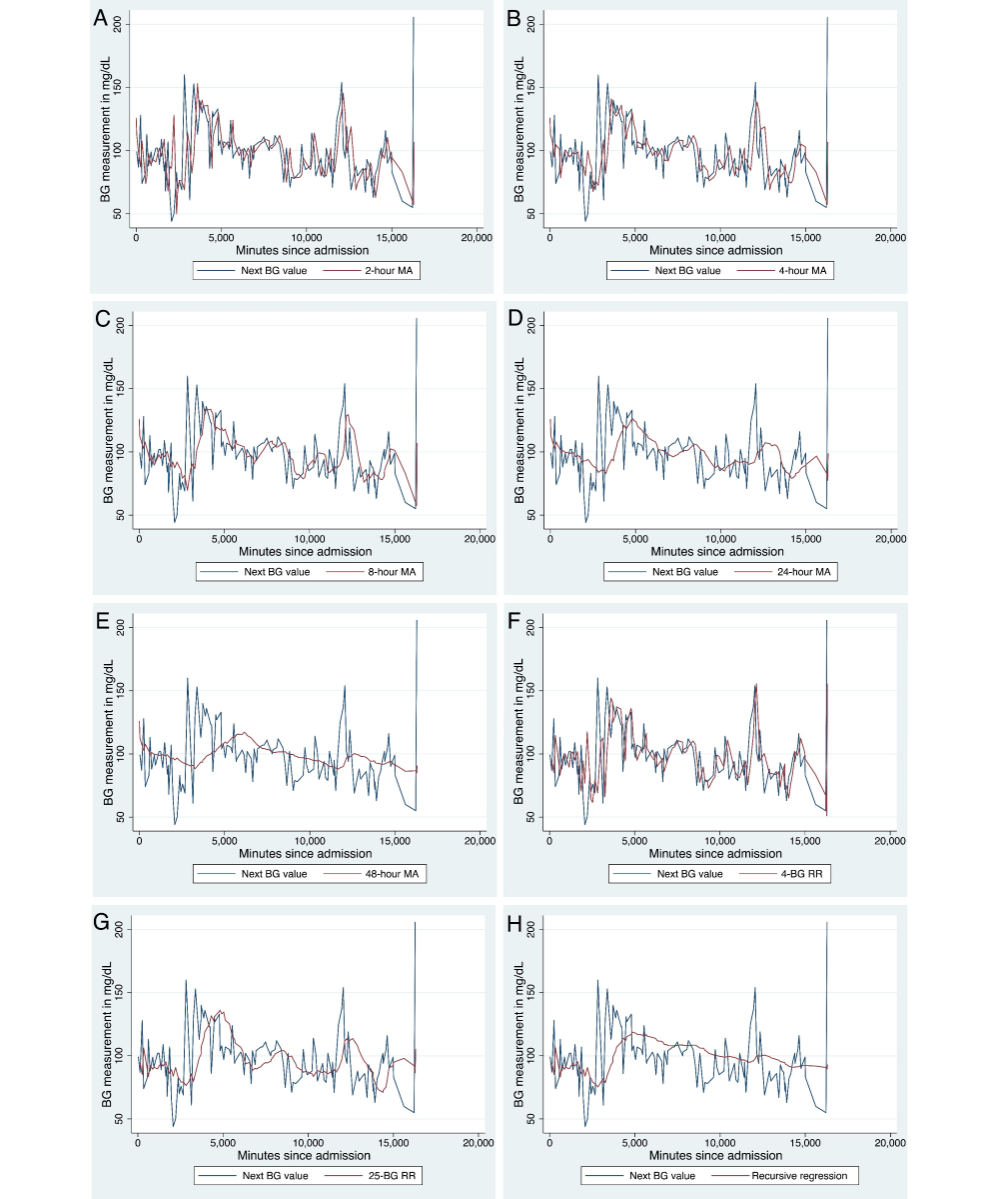
Next blood glucose (BG) measurement and predicted BG measurement throughout an example patient’s admission. A patient’s next BG value (blue line) compared with what would be predicted based on a time series predictor (red line). The time series predictors that were compared with the true next BG value were (A) 2-hour moving average (MA), (B) 4-hour MA, (C) 8-hour MA, (D) 24-hour MA, (E) 48-hour MA, (F) 4-BG rolling regression (RR), (G) 25-BG RR, and (H) recursive regression.

The data set was further divided into categories of CV to determine if BG measurement lability affected predictive accuracy of different time series predictors: *low glycemic variability* if over the past 24 hours the CV was ≤0.15 (404,840 observations), *medium glycemic variability* if over the past 24 hours the CV was >0.15 and ≤0.30 (1,442,328 observations), *high glycemic variability* if over the past 24 hours the CV was >0.30 and ≤0.45 (456,584 observations), and *very high glycemic variability* if over the past 24 hours the CV was >0.45 (132,476 observations). Table 2 shows the Pearson correlation coefficient between the predicted BG value and next BG value of various MA and RR intervals. There was an inverse relationship noted between *R*^2^ and time away from index BG value in the moving regression analyses; for example, the *R*^2^ value of the relationship between the next BG measurement and 2-hour MA predictor was 0.504 and between the next BG measurement and the 36-hour MA predictor was 0.440. The sample-and-hold technique, which is the correlation coefficient between the current and next BG measurement, had the highest *R*^2^ value (*R*^2^=0.529; RMSE=47.16). Furthermore, the *R*^2^ value drops as the category of glycemic variability increases. A comparison between different time series predictors and the next BG measurement for a representative admission is included (Figure 4). Different time series predictors (ie, 2-hour MA, 4-hour MA, etc) that would be calculated with each new BG measurement are plotted with the true value of the next BG measurement. Predictors with longer time horizons (such as the 48-hour MA or 25 measurement recursive regression) have a smoother curve as they represent the overall average of a patient’s BG measurements rather than the most recent BG measurements.

**Table 2:** Pearson correlation coefficient of time series predictors plotted with next blood glucose (BG) measurement.

Model			*R^2^*								
			All observations		Glycemic variability category^a^						
					Low		Medium		High		Very high
**Moving average**											
	30 minutes	0.527		0.684		0.513		0.380		0.368	
	1 hour	0.519		0.683		0.508		0.370		0.348	
	1.5 hours	0.512		0.682		0.502		0.356		0.322	
	2 hours	0.504		0.682		0.498		0.336		0.290	
	4 hours	0.481		0.680		0.480		0.290		0.205	
	8 hours	0.489		0.682		0.495		0.296		0.162	
	12 hours	0.483		0.675		0.486		0.292		0.157	
	16 hours	0.467		0.664		0.469		0.273		0.144	
	20 hours	0.465		0.656		0.466		0.277		0.148	
	24 hours	0.454		0.646		0.453		0.266		0.143	
	36 hours	0.440		0.628		0.438		0.256		0.140	
	48 hours	0.432		0.617		0.429		0.251		0.139	
	60 hours	0.427		0.610		0.423		0.248		0.139	
	72 hours	0.423		0.605		0.418		0.245		0.138	
**Rolling regression**											
	3 observations	0.529		0.679		0.540		0.398		0.370	
	4 observations	0.495		0.672		0.499		0.341		0.321	
	5 observations	0.484		0.670		0.488		0.321		0.298	
	10 observations	0.491		0.667		0.501		0.334		0.257	
	25 observations	0.465		0.647		0.478		0.304		0.199	
	100 observations	0.433		0.609		0.444		0.276		0.177	
	500 observations	0.427		0.601		0.437		0.272		0.175	
Recursive regression			0.427		0.601		0.437		0.272		0.175
Current BG (sample-and-hold)			0.529		0.685		0.515		0.381		0.373

^a^Glycemic variability defined based on coefficient of variation (CV) over the previous 24 hours including the index BG measurement: low (CV≤0.15), medium (0.15<CV≤0.30), high (0.30<CV≤0.45), and very high (0.45>CV). A good *R*^2^ value is >0.75, an acceptable *R*^2^ value is between 0.50 and 0.75, and an inadequate *R*^2^ value is <0.50.

### Performance of BG Predictions With Clinical Covariates

The performance of 4-hour and 24-hour MA, 3-observation and 25-observation RR, and recursive regression BG predictions are compared in the full population, patients with T1DM, and patients with type 2 diabetes mellitus with basal insulin on board (Table 3). In the general patient population, the 4-hour and 24-hour MA performed similarly on Clarke Error Grid analysis, with 94.4% and 94.3% of predictions in zones A and B, respectively. The 3-observation RR had 97.9% of predictions in zones A and B, but only 27.3% of all predictions were in zone A. In the population with type 1 diabetes, <85% of predictions for all models except the 3-observation RR were in zones A and B. In the population with type 2 diabetes, all models had at least 90% of predictions in zones A and B except for the 3-observation RR. When comparing observation-level characteristics of the entire patient cohort with the observations that occurred in Clarke Error Grid zones C to E, we found a higher proportion of misclassifications occurred in patients who were on home insulin (eg, 33.8%, 34.1%, and 31.8% in the 3-observation RR, recursive regression, and 30-minute MA, respectively, vs 20.3% in the entire cohort), patients who were on home steroids (eg, 8.1%, 7.8%, and 7.6% in the 3-observation RR, recursive regression, and 30-minute MA, respectively, vs 6.3% in the entire cohort), and patients who were African American (eg, 37.8%, 39.2%, and 37.6% in the 3-observation RR, recursive regression, and 30-minute MA, respectively, vs 33.8% in the entire cohort).

Table 4 compares the performance of different machine learning models when all time series predictors were used in these algorithms. The Cubist model performed the best (RMSE=44.9, 95% CI 42.8-47.0), and k-nearest neighbors model performed the worst (RMSE=49.4, 95% CI 46.7-50.8), although these differences were not statistically significant. Including non-BG predictors in these machine learning algorithms did not meaningfully improve predictive performance. The best performing unadjusted model was the linear model (*R*^2^=0.561, 95% CI 0.536-0.586), which was statistically significantly greater than the correlation between the 30-minute MA and next BG measurement (*R*^2^=0.527).

**Table 3:** Clarke Error Grid analysis results for different time series analyses by diabetes diagnosis.

Clarke Error Grid zone			Full population (n=2,436,228)											Type 1 diabetes (n=104,115)											Type 2 diabetes^a^ (n=351,252)								
			A^b^		B^c^		C^d^		D^e^		E^f^		A			B		C		D		E		A			B		C		D		E
**Moving average**																																	
	4 hours	0.61		0.33		0.02		0.03		0.00		0.43			0.40		0.09		0.07		0.01		0.51			0.40		0.04		0.05		0.00	
	24 hours	0.59		0.35		0.02		0.03		0.00		0.39			0.43		0.10		0.06		0.02		0.50			0.42		0.04		0.04		0.01	
**Rolling regression**																																	
	3 observations	0.27		0.71		0.01		0.01		0.00		0.26			0.69		0.02		0.03		0.01		0.25			0.71		0.02		0.02		0.00	
	25 observations	0.55		0.41		0.02		0.03		0.00		0.39			0.47		0.05		0.07		0.01		0.49			0.42		0.03		0.05		0.01	
	Recursive regression	0.53		0.42		0.02		0.03		0.00		0.37			0.49		0.05		0.08		0.02		0.47			0.44		0.04		0.06		0.01	

^a^With basal insulin on board at time of blood glucose reading.

^b^A: Values indicate proportion of predicted glucose values that are within 20% of true value.

^c^B: Values indicate proportion of predicted glucose values that are outside of 20% but would not lead to inappropriate treatment.

^d^C: Values indicate proportion of predicted glucose values that are within a range that would lead to unnecessary treatment.

^e^D: Values indicate proportion of predicted glucose values that are within a range that indicates potentially dangerous failure to detect hypoglycemia or hyperglycemia.

^f^E: Values indicate proportion of predicted glucose values that are within a range that would confuse treatment of hypoglycemia for hyperglycemia and vice versa.

**Table 4:** The 5-fold cross-validation statistics of time series predictors used to predict next blood glucose (BG) value in various machine learning models.

	Model A^a^				Model B^b^		
	RMSE^c^ (95% CI)	_*R*_^2^ (95% CI)	MAE^d^ (95% CI)	RMSE (95% CI)		_*R*_^2^ (95% CI)	MAE (95% CI)
_Cubist_	_44.9 (42.8-47.0)_	_0.561 (0.536-0.586)_	_29.3 (28.8-29.3)_	_44.9 (43.2-46.6)_		_0.563 (0.533-0.593)_	_29.3 (29.0-29.6)_
_Linear model_	_44.8 (42.9-46.6)_	_0.562 (0.532-0.592)_	_29.7 (29.1-30.2)_	_46.9 (42.5-51.2)_		_0.526 (0.461-0.591)_	_29.9 (29.2-30.7)_
_Random forest_	_45.4 (43.9-47.0)_	_0.547 (0.521-0.575)_	_30.4 (29.6-31.1)_	_45.2 (42.9-47.4)_		_0.554 (0.535-0.574)_	_30.3 (29.7-30.8)_
_Partial least squares_	_45.4 (43.8-47.0)_	_0.548 (0.512-0.584)_	_30.2 (29.5-30.9)_	_46.0 (44.3-47.6)_		_0.538 (0.502-0.574)_	_30.7 (30.0-31.5)_
_k-nearest neighbors_	_48.7 (46.7-50.8)_	_0.486 (0.469-0.503)_	_32.8 (32.6-33.0)_	_49.4 (46.8-51.9)_		_0.472 (0.439-0.505)_	_33.4 (32.6-34.1)_

^a^Model A: predictor variables in all machine learning models above were 30-minute moving average (MA), 1-hour MA, 1.5-hour MA, 2-hour MA, 4-hour MA, 8-hour MA, 12-hour MA, 16-hour MA, 20-hour MA, 24-hour MA, 36-hour MA, 48-hour MA, 60-hour MA, 72-hour MA, 3-observation rolling regression (RR), 4-observation RR, 5-observation RR, 10-observation RR, 25-observation RR, 100-observation RR, 500-observation RR, recursive regression, index BG measurement, and previous BG measurement.

^b^Model B: all variables included in model A and sex, age, race, diabetes diagnosis, nil per os status, home insulin, home antihyperglycemic medication, glomerular filtration rate, hydrocortisone equivalents on board, basal insulin units on board (units, U), combination insulin units on board (U), concentrated insulin units on board (U), intermediate acting insulin units on board (U), rapid-acting insulin units on board (U), regular insulin units on board (U), and ultra–long-acting insulin units on board (U).

^c^RMSE: root mean squared error.

^d^MAE: median average error.

## Discussion

### Principal Findings

In this retrospective cohort study using a large number of POC and serum glucose observations, we identified the correlation of different time-varying MA and RR predictors of a hospitalized patient’s next BG reading. We found that the most recent BG measurement provides the most predictive accuracy; adjusting for trends or increasing the lookback window negatively affects correlation. Interestingly, the addition of variables associated with glycemic control did not greatly modify the performance of machine learning algorithms that included all the MA and RR predictors, although the machine learning models performed marginally better compared with any individual time series predictor. However, the best performing algorithm in model A (time series predictors only) was the simple linear regression, but the best performing algorithm in model B (time series predictors with additional nonglycemic data) was the Cubist model, suggesting that new information differentially improved different algorithms.

In clinical practice, there is growing interest in developing machine learning algorithms to predict hypoglycemia in the inpatient setting. Although many of the published algorithms use categorical variables [38], consideration should be given to models that quantitatively predict BG, similar to CGM data. Our findings suggest that smaller prediction horizons are correlated more to the next BG measurement compared with longer periods of data, which suggests that clinicians should consider more recent BG measurements when attempting to predict the next BG measurement. Future studies attempting to quantitatively predict BG could create trend arrows based on the current glucose variable to the predicted variable that could be coupled with actionable insulin titration. Using trend arrows to guide insulin dose adjustments in patients who use CGMs has been previously discussed [51-53]. Trend arrows, which would demonstrate the degree of a patient’s BG trajectory (ie, increasing rapidly, increasing slowly, remaining level, decreasing slowly, and decreasing rapidly), may guide titration of correctional bolus insulin doses and daily basal-bolus insulin dosing for hospitalized patients, although such an algorithm would require validation.

### Comparison With Prior Work

There has been interest in using CGM data to predict a patient’s BG over a short horizon of 60 minutes. For example, Gani et al [54] derived a linear autoregressive model that had a 60-minute average RMSE of 12.6 mg/dL, and Zhao et al [55] created a latent variable-based statistical method with an average RMSE of 29.2 mg/dL and 72.1% of BG readings in zone A of the error grid. Recently, deep learning techniques such as a semisupervised deep neural network [56], nonlinear autoregressive neural network [57], and recurrent neural networks [58] have demonstrated improved performance in BG prediction over 30-, 60-, and 90-minute prediction horizons. A recently published review that analyzed 63 studies found that data-based models, which used artificial neural networks and hybrid models, performed better in predicting hypoglycemia and offered promise in applicability and performance [17]. However, most of these studies are limited to small sample sizes of patients with T1DM in a nonhospitalized setting. Previously published glucose prediction in hospitalized patients has focused on predicting future hypoglycemia over the prediction horizon of 24 hours [24,28] and a patient’s admission [32]. Recent studies have predicted future hyperglycemia and hypoglycemia as categorical outcomes [35,36]. Although several recent studies have used machine learning to predict the next category of glucose (ie, hypoglycemic, controlled, or hyperglycemic), there are no studies that have tried to predict the next glucose value as a continuous outcome using electronic health record data alone.

Our study found that the predictive accuracy of MA and RR declined with increasing size of the lookback window. Although BG is generally obtained either every 4 hours or 4 times daily, the 30-minute MA had the highest predictive accuracy based on the *R*^2^ value alone. This finding is likely because most patients did not have 2 BG readings in any 30-minute interval, so the 30-minute MA was equal to the most recent BG reading. Similarly, predictive accuracy drastically declined with increasing glycemic variability. Glycemic variability has been shown to be significantly associated with clinically significant hypoglycemia (BG<54 mg/dL) [59], suggesting that this population warrants the highest need for accurate BG prediction.

A secondary objective was to evaluate how performance accuracy differs when comparing a model using BG data alone with one that includes a broader number of clinical variables that can influence glucose homeostasis. A recent review of BG prediction strategies in patients with T1DM using CGM data found that most published models use CGM data, insulin dosing, and carbohydrate consumption [60]. However, these models have time horizons of up to 1 hour, so it is difficult to distinguish the predictive performance of non-CGM data in models with shorter prediction horizons. Of note, the *R*^2^ of the linear regression model decreased with the addition of demographic and insulin variables, suggesting that these variables worsened predictive accuracy in a linear regression. However, the highest performing model when demographic and insulin variables were included was a Cubist model, which fits a regression model based on a rule that is derived from a collapsed tree structure that is pruned and combined [61]. Interestingly, adding additional covariates, which we expected to explain some of the variability for next BG, resulted in equal or worse fits for the prediction of next BG measurement. Notably, our previous work demonstrated that BG history had the most predictive value in a random forest model [35], which corresponds to these findings that time series variables provide significant predictive value compared with other clinical predictors.

On the basis of the relatively similar performances of the MA and RR, we were surprised how greatly the Clarke Error Grid analysis differed between the MA and RR results. For example, in the full patient population, 61% of the 4-hour MA predictions but only 27% of the 3-observation RR predictions were in zone A. These findings highlight limitations in deciding which performance metrics to report. As described previously, mean squared error and sum of squared errors are the most commonly reported performance metrics in BG prediction [60]. Error-based metrics are limited because they do not identify whether misclassification is occurring during hypoglycemic, euglycemic, or hyperglycemic events [62]. Furthermore, we were surprised to see the difference in Clarke Error Grid performance based on the diagnosis of diabetes.

Our study has several strengths. Notably, we determined the correlation of different time series predictors with the next BG measurement. We also evaluated the predictive performance when all time series predictors were included in machine learning models that included other demographic and clinical parameters. Our analyses were based on a large, diverse sample. Although BG prediction algorithms published in the literature use CGM data, our analysis can be applied to hospitalized patients who do not have access to CGMs.

### Limitations

There are some limitations to our study. We did not have information about insulin doses from total parenteral nutrition formulations, amount of carbohydrates consumed with meals, or designation of BG as either random or fasting. Similarly, measures such as hemoglobin A1c were not included as not all patients have this routinely measured during admission. As we attempted to predict a patient’s next BG measurement based on POC or serum BG readings, the time to next BG reading is not defined like in CGMs. Thus, we were unable to define a discrete prediction horizon as BG samples are not obtained at exact intervals in every inpatient. In addition, much of our analysis was based on the correlation between a BG reading’s next measurement and its associated time series predictors. This analysis has limited predictive value as these time series predictors were not tested on a test cohort of data. The machine learning approaches presented to combat this limitation may be prone to overfitting given the complexity of the models. Although the machine learning models were significantly more predictive than any individual time series predictor, the clinical significance of these findings is uncertain given the only modest increases in *R*^2^ value with the machine learning models. Finally, the time series predictors performed poorly as glycemic variability increased, which is the type of patients that could benefit most for a tool to predict the next glucose value. Similarly, patients with T1DM, who may be more at risk for dysglycemia owing to insulin needs, had no glycemic predictor achieve >45% of predictions in zone A of the Clarke Error Grid.

### Conclusions

To the best of our knowledge, this is the first study to evaluate different prediction models for the value of the next BG measurement using only POC and serum glucose measurements in hospitalized patients. Our results did not rely on data from CGMs and were agnostic to when the patient’s next BG would be measured. We found that BG prediction is highly dependent on the most recent BG observation, with diminishing performance as the lookback window increases. Future prospective studies need to evaluate prediction of BG using such time series models and determine whether quantitative prediction of glucose results in better clinical outcomes compared with previous studies that predict hypoglycemic and hyperglycemic events as binary or categorical outcomes.

## Abbreviations

BG: blood glucose
CGM: continuous glucose monitor
CV: coefficient of variation
MA: moving average
POC: point-of-care
RMSE: root mean squared error
RR: rolling regression
T1DM: type 1 diabetes mellitus
